# 仪器分析综合实验:电感耦合等离子体质谱法测定镓元素

**DOI:** 10.3724/SP.J.1123.2024.04025

**Published:** 2025-03-08

**Authors:** Haijiao ZHENG, Yaming ZHAO, Xiaofeng WU, Hai XU, Qiong JIA

**Affiliations:** 吉林大学化学学院, 吉林 长春 130012; College of Chemistry, Jilin University, Changchun 130012, China

**Keywords:** 综合性实验, 仪器分析, 固相微萃取, 代谢组学, 电感耦合等离子体质谱法, comprehensive experiment, instrument analysis, solid phase microextraction (SPME), metabonomics, inductively coupled plasma mass spectrometry (ICP-MS)

## Abstract

“高山仰止,方知才疏。”为进一步推动新课程标准的落地,本文设计了一个仪器分析综合型实验用于本科生仪器分析实验教学。代谢组学是继基因组学和蛋白质组学之后发展起来的新兴组学技术,是系统生物学的重要组成部分。本论文以了解代谢组学在环境与健康领域中的重要应用为目的,通过查阅文献和分组讨论的环节确定本实验选取的用于镓元素分离富集的样品前处理技术及检测方法。随后,让学生通过实验分析了添加金属抗癌镓类药物后培养的细胞中代谢产物的含量变化,进一步引导学生理解代谢组学在环境与健康领域的广泛应用。本实验结合代谢组学在生理药理学中的重要作用,以X射线光电子能谱仪和热重分析仪等大型精密仪器辅助材料的表征,以电感耦合等离子体质谱法对细胞代谢产物中的金属镓元素进行定性与定量分析。本综合实验由教师选题并引导学生综合利用大型仪器去解决问题,做到以研促教,教研相长,为今后常态化地打造仪器分析综合型实验的高效课堂打下坚实基础。

## Abstract (en)

“Admiring the lofty mountains, one realizes their own lack of talent.” To further promote the implementation of new curriculum standards, a comprehensive instrumental analysis experiment was designed. Metabolomics, an emerging technology developed after genomics and proteomics, is an important part of systems biology. This study aims to explore the applications of metabolomics in the fields of environment and health. Sample pretreatment technology and detection methods for separating and enriching gallium were chosen through a literature review and group discussion. Students then experimentally analyzed the changes in metabolite content in cells cultured with metal-anticancer gallium drugs, which helped them understand the widespread application of metabolomics in the fields of environment and health. Additionally, material characterization was conducted using X-ray photoelectron spectroscopy (XPS) and thermogravimetric analysis (TG), and the gallium metal in metabolites was qualitatively and quantitatively analyzed using inductively coupled plasma mass spectrometry (ICP-MS). During discussions of this experiment, the teacher guided students in using large-scale instruments to solve problems comprehensively, fostering a research-based teaching approach to build a solid foundation for conducting efficient instrument analysis and comprehensive experiments within the classroom in future.

代谢组学是继基因组学和蛋白质组学之后发展起来的新兴组学技术,是系统生物学的重要组成部分。代谢组学的研究目标多是某一生物体组分或细胞在一特定生理时期或条件下的代谢产物,如氨基酸、糖、脂质、金属离子、芳香烃等小分子物质并对其进行定性和定量分析^[1,2]^。仪器分析实验作为化学、化工、环境、药学、生命科学等相关专业的重要实验课,肩负着培养学生动手能力,掌握大型仪器基本操作的任务。该课程的授课对象主要为大二、大三年级本科生及研究生。相比于教法单一、验证性实验较多、实验教程基本有完整步骤的现行仪器分析实验,综合实验将验证性实验与实际应用相关联,实验过程涉及一门课程中的众多知识点和与该课程相关的其他课程的综合运用,在保证学生完成基本实验技能训练的基础上,培养学生创造性思维和综合解决问题的能力。笔者所在教学团队长期承担仪器分析理论课及仪器分析实验课,且具有国家自然科学基金支持的科研成果^[3⇓-5]^。将前沿性的代谢组学科研成果转化成综合仪器分析实验教学的内容,通过“教”与“研”二者有机结合,做到以研促教,教研相长,迈出高质量科研成果的教学转化一大步,促进实施高质量实验课程的改革。

近年来,具有抗癌活性的金属配合物不断被合成出来,如铂类、钌类和镓类等抗癌药物。研究表明,与传统的金属抗癌药物氯化镓相比较,三(8-羟基喹啉)镓(简称GaQ_3_)具有更好的抗癌效果,但仍有一定的毒副作用。因此,在细胞水平上分析金属药物的代谢产物(如金属离子)具有重要意义。目前,测定镓元素的最有效方法集中于电感耦合等离子体光谱法(ICP-AES)和电感耦合等离子体质谱法(ICP-MS)^[6⇓-8]^。ICP-MS具有灵敏度高、检出限低、耗时短等优点。将其用于复杂生物样品分析时,适当的样品前处理能够达到去除复杂基体、富集目标分析物的目的。分离和富集镓元素的前处理方法主要有液-液萃取法、索氏提取法、离子交换法和固相萃取法等^[9⇓⇓-12]^,然而对于镓抗癌药物分析的前处理方法却很少。另外,上述前处理方法存在一些问题,如使用溶剂较多、耗时过长等。2006年,一种基于固相微萃取的新型样品前处理方法被提出,即聚合物柱微萃取(PMME)^[13,14]^。聚合物整体柱具有制备过程简单、通透性好、背压低、传质速率快、比表面积大、耐酸碱、机械稳定性良好等优点。本实验基于团队前期科研成果,由教师完成PMME装置的制备,随后,指导学生使用X射线光电子能谱仪和热重分析仪对材料进行表征以及指导学生完成样品萃取步骤,最后,利用ICP-MS作为检测手段测定添加GaQ_3_培养的细胞中镓元素的含量。

## 1 实验部分

### 1.1 仪器、试剂与材料

ESCALAB QXi X射线光电子能谱仪(XPS,赛默飞世尔,美国); Q500热重分析仪(TG,珀金埃尔默,美国); Thermo iCAP Qc电感耦合等离子体质谱(赛默飞世尔,美国),配置高效四极杆雾化器;LSP01-1A型注射泵(保定兰格恒流泵有限公司);石英毛细管(530 μm o.d.×690 μm o.d.,河北永年锐沣器件有限公司); S400酸度计(梅特勒托利多有限公司); JJ-1型磁力电动搅拌器(江苏金坛市江南仪器厂); Novo Cyte流式细胞仪(安捷伦,美国); ZK-82BB型真空干燥箱(上海实验仪器有限公司)。

甲基丙烯酸缩水甘油酯(GMA,97%)、*γ*-(甲基丙烯酰氧)丙基三甲氧基硅烷(*γ*-MAPS, 97%)、偶氮二异丁腈(AIBN,98%)、乙二醇二甲基丙烯酸酯(EDMA,98%)和磷酸盐缓冲溶液(PBS,20×)购于Aladdin试剂有限公司。二甲基亚砜(DMSO,99.5%)、环丙基溴(C_3_H_5_Br,99%)、对甲基苯磺酰氯(TsCl,≥97%)、八水合氢氧化钡(Ba(OH)_2_·8H_2_O,98%)、氧化钡(BaO,97%)、溴化十六烷基三甲铵(CTAB,99%)、8-羟基喹啉(99%)、硫脲(99%)、*β*-环糊精(*β*-CD,98%)、1,4-丁二醇(99%)、正丙醇(无水级,99.7%)均购于上海国药集团化学试剂有限公司。乳铁蛋白(LF,95%)、乙二胺四乙酸(EDTA,99%)、硫酸亚铁铵(≥98%)、三氯化钾(GaCl_3_,99.99%)均购于Sigma试剂有限公司。细胞培养所需药品和异硫氰酸类荧光素及碘化丙啶(Annex V-FITC/PI)凋亡检测试剂盒(BestBio,中国)均购于生工生物工程(上海)股份有限公司。GaQ_3_按照文献[15]方法合成。实验时用水稀释1000 g/L的Ga(Ⅲ)标准液(Sigma试剂有限公司)。

### 1.2 仪器检测条件

XPS条件 从聚(GMA-EDMA)微萃取柱和聚(GMA-EDMA-*β*-CD-LF)微萃取柱中取出粉末样品各10 mg用于测试。仪器能量扫描范围为1~4000 eV,能量分辨率≤0.45 eV,空间分辨率≤3 μm,灵敏度为1000000 cps,真空度约为2.04×10^-7^ Pa。TG测试条件如下:样品制备与上述XPS样品制备过程相同;仪器升温速率为10 ℃/min,温度范围为室温~800 ℃,吹扫气氛为氮气,电子天平灵敏度为0.1 μg。

ICP-MS条件 射频功率为1250 W,等离子体气体(Ar)流量为14.0 L/min,辅助气(Ar)流量为0.10 L/min,载气(Ar)流量为1.05 L/min,采样深度8.0 mm,取样器和撇油器孔直径分别为镍1.0 mm和0.4 mm,扫描模式为跳峰扫描,停留时间100 ms,每谱峰点数为1,同位素选取^71^Ga。

### 1.3 实际样品处理及微萃取柱的制备(视频演示)

将Molm-13细胞系置于含有10%胎牛血清和100 U/mL青霉素和链霉素的RPMI培养基中,在含5%CO_2_+95%空气的37 ℃潮湿氛围中培养,然后在真空冰水浴中超声5 min(开5 s,关10 s)提取蛋白质。细胞培养过程中每周进行支原体检测。然后将溶液在20000 r/min下离心30 min,上清液在-80 ℃保存以备后续使用。随后,用0、2.5、5、10 μmol/L的GaQ_3_处理Molm-13细胞24 h,使用Annex V-FITC/PI凋亡检测试剂盒进行流式细胞术分析。

通过流动注射泵往毛细管柱中依次通入H_2_O、NaOH(1 mol/L)、H_2_O、HCl(1 mol/L)、H_2_O、丙酮,每种试剂各冲洗20~30 min (流速为0.2 mL/min)。用硅烷化试剂混合液(*γ*-MAPS∶丙酮=4∶6, v/v)充满毛细管内,管两端密封,反应条件为55 ℃,过夜。反应结束后用丙酮冲洗毛细管约1 h(流速为0.1 mL/min),氮气烘干备用。改性剂*β*-环糊精-乳铁蛋白复合物(*β*-CD-LF)的制备按照文献[16]合成,制备过程见图1。由于*β*-CD与Ga(Ⅲ)/GaQ_3_之间具有主客体识别作用,而天然低饱和度的LF除了能够强烈地螯合环境中的Ga(Ⅲ),还能在LF的N-/C-末端形成配合物^[17,18]^。因此,加入改性剂*β*-CD-LF可以明显提高本方法的萃取效率。将所合成的*β*-CD-LF复合物按照不同比例与单体GMA 240 mg、交联剂EDMA 160 mg、致孔剂正丙醇320 mg和1,4-丁二醇160 mg、引发剂AIBN(按质量分数为1%的单体量计算)混合得到不同比例的聚合液,将聚合液超声30 min后通入氮气10 min,得到的混合液通入处理好的毛细管中,在60 ℃下反应24 h。最后,反复冲洗毛细管以除去未反应物。

**图1:**
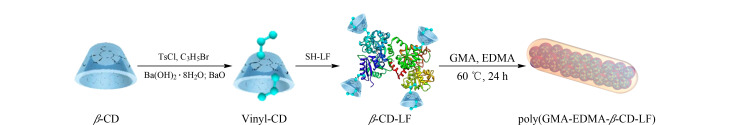
聚(GMA-EDMA-*β*-CD-LF)微萃取柱的制备过程

### 1.4 微萃取流程

PMME装置由注射泵和5 mL注射器组成。每台注射泵可配置两个注射器同时使用。将注射器的针头部位替换成制备好的微萃取柱,所有的溶液均用0.45 μm的滤膜过滤。萃取过程分为预处理、上样、冲洗、排空和解吸5个步骤,具体操作如下:(1)用甲醇在0.05 mL/min的流速下冲洗整体柱5 min; (2)将0.8 mL pH为6.5的样品溶液在0.08 mL/min的流速下通过微萃取柱;(3)用0.5 mL稀释的PBS在相同的条件下冲洗杂质以及未吸附的标准物质;(4)用空注射泵排空整体柱内残留的液体;(5)取0.05 mL 0.1 mol/L的盐酸溶液,在0.03 mL/min的流速下冲洗吸附在微萃取柱上的被吸附物质,收集解吸液,用于ICP-MS分析。

## 2 结果与讨论

### 2.1 微萃取柱的表征

本实验对微萃取柱的热稳定性进行了评价,在氮气氛围下以10 ℃/min的速率从室温升温到800 ℃,结果如图2所示。与未经修饰的微萃取柱(聚(GMA-EDMA)微萃取柱)对比,修饰后的微萃取柱(聚(GMA-EDMA-*β*-CD-LF)微萃取柱)在102.5 ℃处有热量损失,这可能是由于改性剂分解导致物象发生变化,275 ℃处的热失重为整体材料的分解,约450 ℃后成一条直线,此时材料分解较为完全。由于本文所涉及萃取实验均在室温下完成,实验证明,改性前后的微萃取柱在室温下均具有较好的热稳定性,因此,所制备的微萃取柱满足后续实验萃取条件所需。

**图2:**
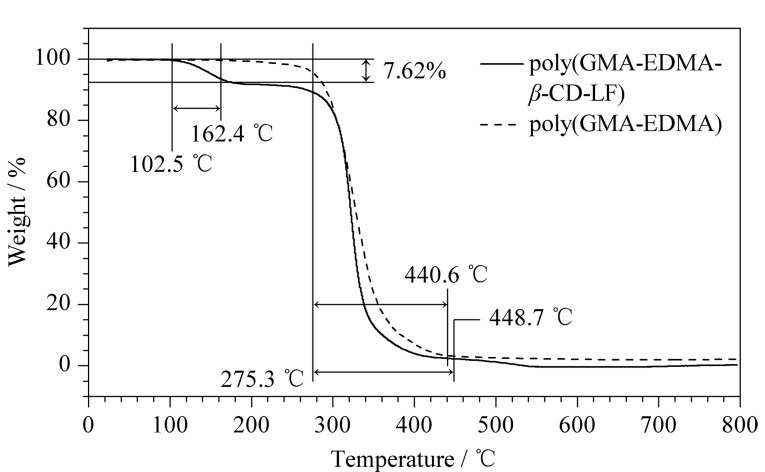
聚(GMA-EDMA)微萃取柱和聚(GMA-EDMA- *β*-CD-LF)微萃取柱的热重分析图

随后,对所合成的微萃取柱进行元素分析测定实验,结果如图3所示,与未经修饰的微萃取柱相比,修饰后的微萃取柱在710.7 eV和164.7 eV处分别出现了Fe 2*p*_3/2_和S 2*p*的峰,并且修饰后的微萃取柱中碳元素和氧元素的含量分别有所增加,其中,碳元素含量从50.6%增加至58.3%,氧元素含量从30.8%增加至40.2%,以上结果说明改性剂成功地键合于材料中。

**图3:**
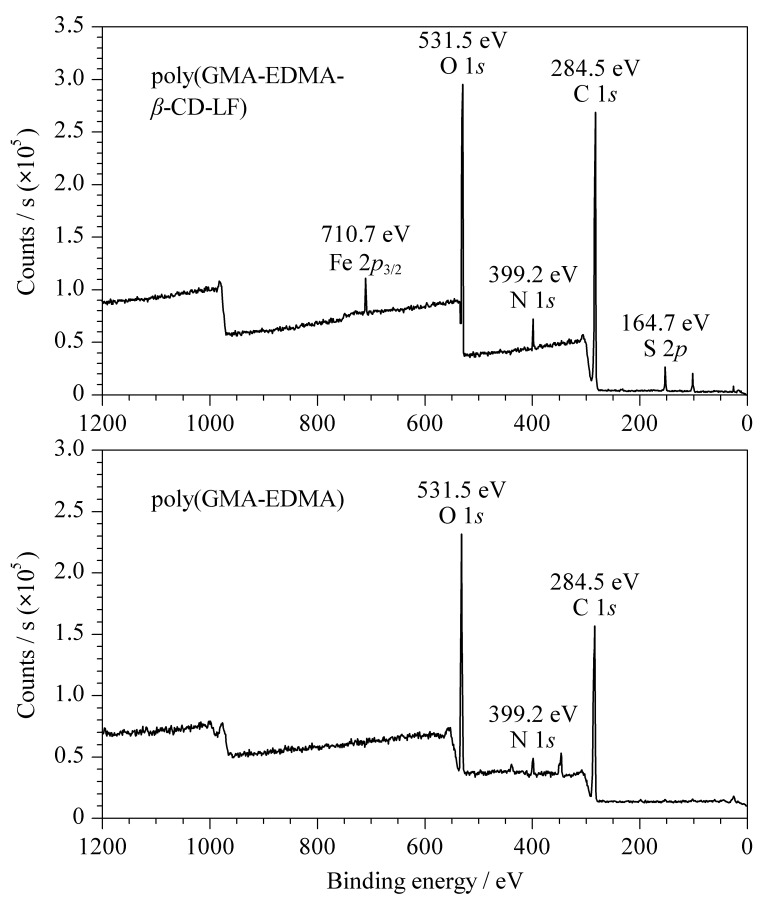
聚(GMA-EDMA)微萃取柱和聚(GMA-EDMA-*β*-CD-LF)微萃取柱的X射线光电子能谱图

### 2.2 PMME条件优化

由于pH值是影响微萃取柱吸附能力的一项重要因素,待测物在溶液中的存在形式与pH值密切相关。实验讨论了pH 2.0~12.0范围内微萃取柱对目标待测物的吸附效率。结果表明,在pH大于7.0时,Ga(Ⅲ)和GaQ_3_的吸附随pH的升高而逐渐降低,主要原因是金属离子在较高的pH条件下易水解。因此,本实验选择pH 6.5为最佳介质酸度。随后,学生参与设计L_9_(3^4^)正交实验,并由学生独立完成各项萃取条件的优化。实验采用L_9_(3^4^)正交实验法考察了样品体积(0.7~0.9 mL)、样品流速(0.07~0.09 mL/min)、解吸液体积(0.03~0.05 mL)以及解吸液流速(0.03~0.09 mL/min)。9组实验数据每组分别平行测定3次,对得到的数据进行方差分析与极差分析,得到最优化的萃取实验条件:样品体积为0.8 mL,样品流速为0.08 mL/min,解吸液体积为0.05 mL,解吸液流速为0.03 mL/min。结果见表1。

**表1:** L_9_(3^4^)正交实验设计及结果(*n*=3)

No.	Sample volume/ mL	Sample flow rate/ (mL/min)	Eluent volume/ mL	Eluent flow rate/ (mL/min)	Mass concentration^*^/ (μg/L)
1	0.7	0.07	0.03	0.03	55.45
2	0.7	0.08	0.04	0.04	56.34
3	0.7	0.09	0.05	0.05	57.43
4	0.8	0.07	0.04	0.05	57.94
5	0.8	0.08	0.05	0.03	58.25
6	0.8	0.09	0.03	0.04	55.21
7	0.9	0.07	0.05	0.04	58.10
8	0.9	0.08	0.03	0.05	56.43
9	0.9	0.09	0.04	0.09	57.94

* Mean mass concentration of triplicate analysis.

### 2.3 方法评价及性能分析

在最优化条件下进行方法评价和性能考察。首先,单个样品的萃取时间约为20 min,包括样品上样时间、清洗时间和解吸时间依次为10、5和1.6 min,样品pH调节和微萃取柱条件设置约为3 min。其次,采用标准曲线法定量,Ga(Ⅲ)的线性范围为0.01~20 μg/L,线性回归系数(*R*)为0.9990。随后,以3倍信噪比(*S/N*)计算Ga(Ⅲ)的检出限(LOD)为2.0 ng/L。以经过微萃取柱萃取与未经萃取的工作曲线的斜率之比来计算微萃取柱对目标分析物的富集倍数,得到方法对Ga(Ⅲ)的富集倍数为29。最后,选取0.1 μg/L的Ga(Ⅲ)样品进行分析,得到Ga(Ⅲ)的日内精密度为2.9%(*n*=3),日间精密度为3.1% (*n*=5)。本实验所采用的实验方法操作简单,无需复杂仪器,有机溶剂用量少,时间短,更加适合用于Ga(Ⅲ)的检测。

### 2.4 实际样品分析

为了验证改性后的微萃取柱用于实际样品分析的可行性,在Molm-13细胞中添加0、2.5、5、10 μmol/L的GaQ_3_进行药物培养实验。细胞毒性实验及凋亡测试结果见图4,引导学生观察加入不同剂量GaQ_3_对细胞活性的影响。随后,学生将由教师处理后的细胞样品经PMME-ICP-MS方法测定样品中的Ga(Ⅲ),实验结果列于表2。结果表明经*β*-CD-LF改性后的微萃取柱可用于复杂生物样品中Ga(Ⅲ)的检测。此外,分析表2中的数据结果可知,本方法所检测到细胞中Ga(Ⅲ)代谢产物的含量随着GaQ_3_在Molm-13细胞样品中加入量的递增而逐渐增加,此结果有助于学生直观地理解代谢组学在检测环境暴露引起的体内代谢变化中的应用,进一步引导学生理解代谢组学在环境与健康领域的应用。

**图4:**
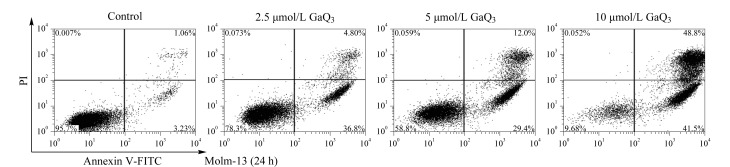
细胞毒性及凋亡测试结果

**表2:** Molm-13细胞中GaQ_3_代谢产物分析(*n*=3)

Sample	GaQ_3_ added/(μmol/L)	Ga(Ⅲ) found/(μg/L)
1	0	N.D.
2	0.5	0.111±0.002
3	1	0.121±0.002
4	2.5	0.223±0.005
5	5	1.178±0.004
6	10	4.142±0.002

N.D.: not detected.

## 3 实验的组织实施及教学反思

### 3.1 实验组织实施及教学总结

本实验通过分组任务的方式完成,每组学生需用时6学时,其中,实验准备阶段1学时,实验操作4学时,结果分析及总结1学时。

实验准备阶段:(1)教师引导学生查阅相关文献;(2)设计实验并细化实验步骤;(3)播放实际样品准备及微萃取柱制备的教学视频并加以讲解。在实验准备阶段,教师从实际生活中的问题出发,培养学生综合思考及通过查阅文献解决问题的能力。

实验进行阶段:将学生分为两人一组,共4组,给每组分配相关实验内容,4组内容分别设置如下:(1)配制标准溶液并采用L_9_(3^4^)正交实验法对影响萃取效果的因素进行优化;(2)使用热分析仪和X射线光电子能谱仪对微萃取柱材料进行表征;(3)使用电感耦合等离子体质谱仪测定标准曲线及计算检出限等;(4)实际样品检测及数据处理。在实验进行阶段,教师将一个完整的大型综合实验分成若干个小型实验,通过团队合作的方式让学生分别完成,其中,单个实验的设置即独立又关联,整体实验过程具有关联性和延续性。这样的设置可以很好地培养学生的独立思考能力和团队协作精神。

结果分析及总结阶段:在小组内探讨实验结果的准确性和实用性;在教师的引导下,学生探讨本实验在实际生产生活中可能的应用,以此培养他们的发散思维和创新思维。

### 3.2 实验问题反馈及注意事项

(1)本实验微萃取柱的合成时间较长,该部分实验由教师提前完成,并录制成视频在课堂演示,在课堂播放视频时教师应配合说明讲解,与学生多进行互动讨论,调动学生的积极性;(2)本实验微萃取流程步骤较多,应按实际操作情况进行学生分配;(3)本实验运用热重分析仪、X射线光电子能谱仪和电感耦合等离子体质谱仪等多种大型仪器,在仪器使用前,学生应独立预习相关仪器说明,教师授课时应对学生充分讲解仪器原理、仪器使用及注意事项,在学生使用仪器时,应全程陪同,从旁指导;(4)实验开展过程中,可能出现微萃取柱堵塞的情况,出现此现象的原因主要是因为完整的萃取流程过后没有及时用溶液清洗微萃取柱,因此,使用时应注意强调微萃取柱的清洗;(5)对于本次实验课程中教师团队观察到的问题,我们提出了细化的解决方案。 例如,教师发现小组内各学生在实验中的参与度不尽相同,我们计划在后续的教学中增加组内评分环节,以求更客观、全面地评价每位学生。

## 4 结语

本实验通过结合固相微萃取技术和电感耦合等离子体质谱仪对镓标准品及细胞代谢产物中的镓元素进行了测定。本实验实现了包含样品表征、萃取实验、大型仪器操作及数据采集分析等步骤的一体化研究过程。通过X射线光电子能谱仪和热重分析仪对微萃取柱进行了表征。通过L_9_(3^4^)正交实验对影响萃取效果的实验条件进行了详细优化。经萃取后的样品通过电感耦合等离子体质谱仪进行镓元素含量的测定及方法学验证。本综合实验引导学生从实际问题出发,调动学生主观能动性,运用所学知识找到解决问题的方法。培养了学生的基本实验技能和团队合作精神,为从事科研工作打下良好的基础。最后,本文所设计的仪器分析综合实验,得到了学生们的积极评价,如“通过本课程了解了代谢组学在环境与健康领域的应用”“分组合作方式较为灵活有趣”等。综上,通过本文所设计的仪器分析综合性实验,达到了构建从“科研水平提高促教学质量”到“教学质量提高促学生水平”到“学生水平提高促科研成果”的教研双边关系的良性循环的目的。
